# The effect of personalized mobile health (mHealth) in cardiac rehabilitation for discharged elderly patients after acute myocardial infarction on their inner strength and resilience

**DOI:** 10.1186/s12872-024-03791-5

**Published:** 2024-02-19

**Authors:** Shahin Salarvand, Farzad Farzanpour, Hasan Ahmadi Gharaei

**Affiliations:** 1 Hepatitis Research Center, Faculty of Nursing and Midwifery, Lorestan University of Medical Sciences, Khorramabad, Iran; 2 Cardiovascular Research Center, Shahid Rahimi Hospital, Lorestan University of Medical Sciences, Khorramabad, Iran; 3 Student Research Committee, Lorestan University of Medical Sciences, Khorramabad, Iran; 4 Torbat Heydariyeh University of Medical Sciences, Torbat Heydariyeh, Iran

**Keywords:** Mobile Health, Cardiac Rehabilitation, Elderly patients, Myocardial infarction, Inner strength, Resilience

## Abstract

**Introduction:**

Given the importance of promoting self-care and quality of life for discharged elderly patients after acute Myocardial Infarction(MI), It is necessitated we conduct interventions to promote these items. This study was conducted to determine the effect of mHealth-Cardiac rehabilitation (CR) on the inner Strength and resilience of elderly patients with MI after discharge from the hospital.

**Methods:**

The present study was a randomized controlled trial that was conducted on 56 Elderly patients with myocardial infarction were discharged from the heart departments. In the intervention group after the patient’s discharge, the patients were contacted twice a week for one month and the necessary training and support were given online. To gather data, the Mini-Mental State Examination (MMSE), the demographic and clinical characteristics questionnaire, the inner strength scale (ISS), and the Connor-Davidson Resilience Scale (CD-RISC) were completed pre- and post-intervention. The data analysis was done by SPSS16.

**Results:**

This study showed the mean resilience and inner strength scores before and after the intervention in the control group had no statistically significant difference(P˃0.05). There was a significant increase in the mean resilience and inner strength scores in the intervention group after the intervention (*P* ≤ 0.001).

**Conclusion:**

The results of this study showed that mHealth as a kind of telenursing nursing has a significant effect on both variables of inner strength and resilience of post-discharge elderly patients after acute myocardial infarction. This means that using mHealth for these patients could increase the inner strength and resilience of the elderly discharged after myocardial infarction. Therefore, through using this method, elderly patients’ self-care ability and quality of life could be increased.

**Supplementary Information:**

The online version contains supplementary material available at 10.1186/s12872-024-03791-5.

## Introduction

With the ever-increasing elderly population worldwide, the prevalence of myocardial infarction is increasing [1]. Myocardial Infarction(MI) has a debilitating and paralyzing nature that severely affects the quality of life (QoL) of affected patients [2]. Considering the psychosocial vulnerability of the elderly [3], a heart attack is considered as a stress affecting the QoL [4], approaching death, and experiencing the need for help and more concern [3].

QoL is one of the predictors of the prevalence of disability and mortality in affected patients [2, 5]. On the one hand, the QoL of myocardial infarction patients is not optimal and they need nursing attention to improve their QoL [2], on the other hand, age is considered as one of the important predictors of general QoL [1, 6–8]. The persons aged 60 years or over are defined as elderly Iran [9]. Given the high number of elderly people in myocardial infarction patients, training and empowering them in self-care and improving their QoL is very important [10]. These patients enter the cardiac rehabilitation(CR) stage after discharge. CR teaches and encourages self-care [11, 12] and promotes QoL [13] Promoting self-care behaviors can help patients control their lives and cope with the complications of their disease, which increases the quality of life in these people [14]. The concept of quality of life is so important that recently the perceived quality of life of patients is measured as the first factor in the effectiveness of any cardiac rehabilitation program [15]. The main components of CR are as follows; Physical activity, behavior change, control of risk factors, nutrition counseling, psychosocial support, and education [16, 17]. Despite the recognized benefits of CR, referral and participation rates remain stubbornly low. More than 80% of patients eligible for CR do not participate [18]. Therefore, nursing interventions extending into rehabilitation provide an opportunity to enhance the QoL of patients for CVDs [19, 20].

Given the burden of cardiovascular diseases, as a chronic disease increasingly demands that patients take more responsibility for their self-management [10], Studies have shown that two predicting factors, improve QoL and increase self-care ability in stressful life events such as MI, are resilience and inner strength [7, 21]. They are protective elements that help an individual cope with stressful events more effectively [22, 23].

Inner strength is one of the strongest predictors of QoL in the elderly. It is an important resource related to aging, disease management, and health [24]. Thus, higher degrees of inner strength have been significantly associated with better health-related QoL [25, 26], and health-promoting behaviors [27]. Desired inner strength means stability, firmness, connection, and excellence. People can withstand adversity, endure pain and suffering, and experience a good life despite difficult circumstances [28]. Among elderly patients with chronic conditions, a low degree of inner strength has been reported. In addition, decreased inner strength is associated with poorer mental health [26] and self-rated health [29].

The resilience of a process is defined as the ability to adapt successfully to threatening conditions [30–32]. It is the capacity to respond positively to stressful events in life [33–35]. Also, resilience can play a strengthening role in the body’s immune system and is the opposite of vulnerability [36]. Resilience in patients with chronic diseases helps patients recognize the changes in their lives and encourages them to actively participate in treatment that can help them return to a healthy life [37]. Several factors play a role in increasing resilience, including genetics, environment, and education [36]. Cardiovascular changes in the elderly have an adverse effect on their QoL, resilience, and longevity [38]. Two studies showed patients with coronary artery disease had lower scores than the resilience mean score [30, 39]. After a MI, one of the important goals of the patient to participate in the CR program is to improve the way of life and adaptability and QoL in the elderly [40]. Learning self-care behaviors can lead people to maintain health, which increases their adaptation to illness [41]. So self-efficacy is a predictor of resilience in the elderly [42].

Poor participation rates, particularly by older adults [43] with their vulnerability and social support issues [44, 45], in conventional CR programs led to the development of home and digital-based interventions [46, 47]. Digital health-based care delivery offers an opportunity to redesign and improve post-discharge care thanks to innovations in telecommunication technologies (e.g. remote CR) [48]. Accommodating CR at home reduces wait times, enables flexible patient participation schedules, and eliminates costs and the need for travel [46, 47]. Patient education with technology can help people with cardiovascular diseases(CVDs) modify their risk factors [49]. and promises to further improve the quality and experience of cardiovascular care [50]. Mobile health(mHealth), the delivery of medical practice by mobile devices [51], has enabled multiple avenues for remote CVDs management. In developing countries, mHealth provides a cost-effective and accessible tool to bridge the health inequality gap and improve chronic disease care [52]. Older adults represent a highly diverse population with a high CVDs burden and have the potential to benefit from interventions that utilize mHealth [53]. Post-MI Home-CR is cost-effective and may be preferable in very elderly and low-risk patients [54].

Teaching and supporting self-care/self-management should be a core activity in our healthcare system [55]. mHealth interventions aimed at self-monitoring of chronic conditions have shown improvement in reducing harmful behaviors [56]. Paying attention to the level of self-efficacy, inner strength, and resilience increases the QoL in heart patients and can have an important effect on their recovery and health [57]. However, most studies on enhancing resilience lack representative samples and pre- and post-intervention evaluations [58]. The review of related literature showed there is a paucity of knowledge about the effect of the personalized mHealth in cardiac rehabilitation for discharged elderly patients after MI on their inner strength and resilience. Gholami, et al., ‘s study, as quasi-experimental research in assessing the effect of in-person education on inner strength and patient activation, reported there was no significant relationship between self-management support programs intervention and CVD patients’ inner strength [59]. Other studies showed the effect of CR on improving QoL in Patients with CVDs [13], the successful behavioral change through short-message service (SMS) via mobile telephone [60, 61], and mHealth can improve heart failure patients’ self-care [62].

On the one hand, the rehabilitation and education of the elderly suffering from MI should be based on the condition of the elderly and their personal needs [3] which can be associated with increasing accessibility and satisfying the unique preferences of patients [63] and on the other hand, some patients may find mHealth-based interventions challenging [47]. According to the relatively large number of studies on the effectiveness of remote CR [64–67], and the role of smart cell phones in patients’ lives and promoting their capacities for self-care [62, 68], mHealth makes an opportunity to improve chronic condition management since mobile phones are so commonly applied, easily accessible, widely accepted, and affordable [61]. The effect of these mHealth-CR educational interventions in improving predisposing factors of self-care, including inner strength and resilience, is unknown. This study was conducted to determine the effect of mHealth-CR on the inner strength and resilience of elderly patients with MI after discharge from the hospital.

## Methods

### Study design

The present study was a parallel randomized controlled trial.

### Participants and setting

**Fig. 1:**
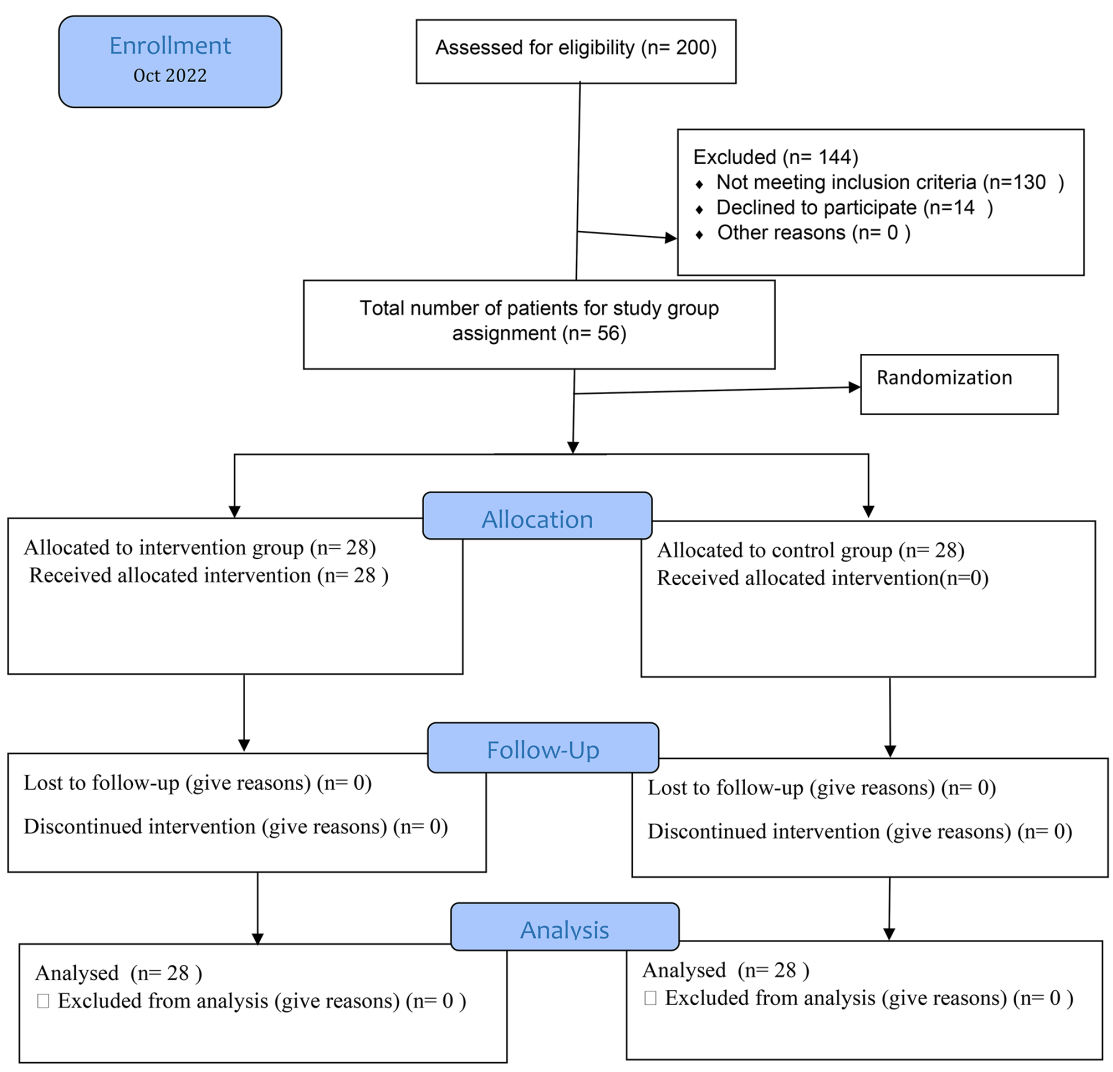
Flow chart of participant enrollment, allocation and data analysis

This study was conducted in the heart departments of teaching hospitals affiliated with Lorestan University of Medical Sciences, Iran. Elderly patients diagnosed with myocardial infarction who were discharged from the heart departments, Shahid Madani and Shafa hospitals, after applying the inclusion criteria, were randomly assigned to the control and intervention groups by the researcher(S.S.). In the pre-test, both the intervention and control groups completed the standard questionnaires of inner strength and resilience, despite the convenience sampling, the allocation of samples in the groups was done with the block random method. For this purpose, first, 14 blocks containing 4 people were designed. Samples were arranged inside the blocks in turn, so that we placed sample 1 in block1, sample2 in block2, …sample 4 in block4, sample 5 in block1,…. then the blocks were numbered and according to the table of random numbers, the block number was selected. the blocks and samples were placed in the intervention or control group based on the arrangement of the blocks and entered into the study. The experimental group was subjected to mHealth nursing care sessions online, while the control group received only training and routine follow-ups(Fig. 1).

The research population of this study was elderly patients with acute MI during discharge from hospitals in Khorramabad city. Including criteria include the following; Age 60 and above, Post-discharge elderly patients with MI, having family caregivers, Elderly or their caregiver access to a smartphone, having no debilitating disease, Having the ability to answer the phone and video chat, and having a minimum EF of 35%. Exclude criteria as follows; drug addiction due to probably non-compliance with recommendations, disturbance in the sense of sight and hearing, and having a cognitive problem according to the mini mental state examination scale(MMSE).

### Sample size

The desired sample size was estimated by considering the error of 1% and the statistical power of 90%, and also by using the standard deviation of the resilience score of the intervention group (δ1 = 13.33) and the control group (δ1 = 9.67) of 23 people in each group [69].

$$\begin{array}{l}n = \frac{{{{(Z1 - \frac{\alpha }{2} + 1 - \beta )}^2} + (\delta {1^2} + \delta {2^2})}}{{{{(\mu 1 - \mu 2)}^2}}}\\n = \frac{{{{\left( {3.85} \right)}^2}{{\left( {13.33} \right)}^2} + {{\left( {9.67} \right)}^2})}}{{{{(68.55 - 55.32)}^2}}} = \frac{{4019}}{{175.03}} = 23\end{array}$$

Taking into account 20% probable attrition, it increased to 28 people in each group, and the total sample size was considered equal to 56 people in both groups.

### Intervention

#### Orientation and recognition stage

After receiving the code of ethics, obtaining a randomized clinical trial code; Id: IRCT20220530055029N1, first trial registration date: 20/07/2023, Trial Id: 63,986, and the letters of introduction from the Research Vice-Chancellor of Lorestan University of Medical Sciences, this study was conducted in two stages: Designing of educational content (Appendix 1) and Implementation of online training of this content with randomized clinical trial method. First, the educational content was compiled by reviewing the related literature and clinical guidelines, and in a survey with eight experts, it was proposed and approved by them in terms of feasibility, relatedness, comprehensibility, and usefulness. The degree of feasibility, relatedness, comprehensibility, and usefulness of each action was given a score of 1–9. Finally, each of the measures was rated as “appropriate”, “uncertain” or “inappropriate”, based on the scores. So the average score was 1–3 as inappropriate, 4–6 as uncertain, and 7–9 as appropriate. Then, the actions that scored 7 or above were included in the educational content [70], and all actions were evaluated as appropriate. And then this content was taught to the elderly with MI through video chat. The tools used include resilience and inner strength measurement tools that were completed before and after the intervention in both groups.

#### Reinforcing and engaging stage

Both groups of patients underwent routine center-based CR, and to prevent contamination, the control group visited the rehabilitation center on odd days and the intervention group visited the rehabilitation center on even days. The researcher(F.F.) interacted with the patients in person at the cardiac rehabilitation center to complete the questionnaire. The method of blinding depends on the type of selective intervention [71]. In our study, both of patients and the statistical analyzer were blinded throughout the entire process. The patients did not know whether they belonged to the intervention or control group. The statistical analyzer was unaware of the objectives of the study and the random distribution of patients in the studied groups.

### Monitoring and follow-up stage

After receiving a letter from the vice-chancellor of treatment of Lorestan University of Medical Sciences, referring to Khorramabad hospitals, a list of the names of elderly patients after acute MI was received. First, the samples were selected based on the inclusion criteria, and according to the MMSE scale, none of the samples had cognitive problems to be excluded from the study (Fig. 1). After determining the samples, mhealth sessions were implemented according to the clinical guidelines for follow-up care in patients with acute MI. In the intervention group after the patient’s discharge, the researcher(F.F.), a MSc gerontology nursing student, contacted the patients twice a week for one month according to the previous coordination between 4 and 7 p.m., and the necessary training and support were given online. The contact time of the researcher could be changed depending on the desire of the patient or his family caregiver to get a better answer for each patient, and it was determined by the patient himself, and if necessary, the patient was given counseling. In case of video chat internet connection failure, the training session was repeated. During the online communication, if a potential problem was observed, the patient’s doctor was informed and the patient was guided to seek immediate care and refer to the emergency room or contact the doctor.

It should be noted that to fully fulfill the supporting role of the nurse, the communication channel with the patient was open and the patient would call in case of a specific problem outside of the necessary training and the need for further consultation. The researcher(F.F.) has either arranged an additional meeting or consulted the patient over the phone to solve the patient’s problem. In addition to a nurse call, patients can call the researcher from 8:00 a.m. to 8:00 p.m. Of course, the patients were not limited to this time frame, and some of them called whenever needed. If necessary, the patient was referred to the relevant specialist. Also, the contact number of the researcher(FF) was given to the patient and his caregiver in case of a problem. The educational techniques were lecturing, question, and answer. In addition, the researcher was addressing the personalized patient concerns in each session.

The control group did not receive online counseling and only received the same routine CR at the time of post-discharge. In the post-test, to contact the patients one month after the completion of the online sessions, coordination was implemented and the standard questionnaires of inner strength and resilience were distributed between the two experimental and control groups after distance nursing training. Finally, the data was collected and analyzed using SPSS 16 software.

### Measures

Four tools used in this study included as follows:

The pre-study assessment part; The Mini-Mental State Examination(MMSE): This scale is effective as a screening tool for cognitive impairment in older, community-dwelling, hospitalized, and hospitalized adults. It is an 11-item scale that tests five domains of cognitive functioning: orientation, registration, attention and calculation, recall, and language. The maximum score is 30. The cut-off point is 23. A score of 23 or less indicates cognitive impairment. It only takes 5 to 10 min to use, making it practical for frequent and routine use [72]. This scale has been psychometrics tested in Iranian society [73, 74] and used in many studies in Iran, and its reliability and validity have been proven [75, 76].

The first part; demographic and clinical characteristics: This section includes the patient’s characteristics (age, gender, level of education, occupation) and clinical characteristics of the patient (current diseases of the patient, history of MI, discharge date, ejection fraction rate), contact phone number (Table 1).

The second part; the inner strength scale(ISS) was developed and tested by Roux, Luis, and Dingli in 2003 [77]. This questionnaire has 27 items, which are graded on a 5-point Likert scale from completely agree 5 to completely disagree 1. The minimum score for this tool is 27 and the maximum score is 135, and a higher score indicates higher inner strength. The cut-off point is 54, so the scores 27 to 54 are considered weak inner strength, and 54.01 to 135 are considered medium and higher [78, 79]. This scale has been used in several studies in Iran and its validity and reliability has been confirmed [80, 81]. At present study, the validity was proven by a survey from five faculty members and confirmed, and the Cronbach’s alpha and test-retest were applied to confirm the inner strength scale’s reliability with 0.84 and 0.78 respectively.

The third part; The Connor-Davidson Resilience Scale (CD-RISC): Resilience questionnaire was prepared by Connor and Davidson (2003) by reviewing the research sources of 1979–1991 in the field of resilience [82]. This scale is the most widely used scale of resilience [83]. CD-RISC contains 25 items, which are rated on a five-point Likert scale and range from 0 (“Not true at all”) to 4 (“True nearly all the time”). Possible scores thus range from 0 to 100 [82]. The higher this score is, the higher the intensity of the individual’s resilience, and vice versa [83]. In the present study, the cutoff point for this questionnaire is 50 points. In other words, a score higher than 50 indicates people with desirable resilience. This instrument has been applied in many studies [32, 84] and its psychometrics has been conducted among the elderly [85], the general population [86, 87], and other specific populations in Iran [88–91].

### The analysis of information and use of statistical methods

The data obtained using the Smirnov-Kolmogorov data normality method and descriptive indices (mean and standard deviation as well as frequency and frequency percentage, graphs) and statistical inference such as independent t-test and paired t-test to determine the mean difference between groups in the pre-test and post-test stages were analyzed. These analyses were done at the error level of 0.05 with the help of SPSS version 16 software.

## Results

The findings of the demographic characteristics of the elderly showed that the two groups were homogeneous and had no statistically significant difference. The average age in the intervention and control groups was 73.14 (SD = 4.36) and 73.50 (SD = 5.59), respectively. The number of men and women in the intervention and control groups did not differ from each other. Other information is provided in Table 1.

**Table 1:** Frequency distribution of demographic characteristics of the elderly in two intervention and control groups

Variable	grouping	Intervention group(*N* = 28) Mean ± SD	Control(*N* = 28) Mean ± SD
Age	60≥	73.14 ± 4.36	73.5 ± 5.59
BMI		27.02 ± 0.90	28.79 ± 1.28
Gender	Male	17(51.5%)	16(48.5%)
	Female	11(47.8%)	12(52.2%)
Education	Illiterate	14(66.7%)	7(33.3%)
	literate	14(40%)	21(60%)
Occupation	Housekeeper/ Farmer	17(53.1%)	15(46.9%)
	Retired/ Non-employee job	11(45.8%)	13(54.2%)

The results showed that the mean resilience score before the intervention and after the intervention in the control group was not statistically different (*P* = 0.18). However, the mean resilience score before and after the intervention in the intervention group was 51.71 ± 4.10 and 67.71 ± 11.86 respectively, which was statistically significant (*P* = 0.001). In addition, the results showed that the mean score of inner strength in the control group, before and after the intervention, had no statistical difference (*P* = 0.71), but the mean score of inner strength in the intervention group, before the intervention (88.00 ± 4.69). There was a significant difference with the post-intervention (97.68 ± 4.53) (*P* = 0.001) (Table 2).

**Table 2:** Intragroup comparison of Resilience and inner strength in control and intervention groups before and after the intervention

Variable	Groups	Before Intervention Mean ± SD	After Intervention Mean ± SD	Paired T-Test	*P*-value
Resilience	Control	49/86 ± 20/70	50.07 ± 20.23	1.36	0.18
	Intervention	51.71 ± 4.10	67.71 ± 11.86	7.93	< 0.001
Inner strength	Control	85.50 ± 9.15	85.46 ± 8.96	0.37	0.71
	Intervention	88.00 ± 4.69	97.68 ± 4.53	11.94	< 0.001

The results showed that before the intervention, the mean resilience score in the two control groups (49.86 ± 20.70) and the intervention (51.71 ± 4.10) had no statistical difference (*P* = 0.64). However, after the intervention, the mean resilience score in the control group was 50.07 ± 20.23, and in the intervention group was 67.71 ± 11.86, which was statistically significant (*P* = 0.001). Moreover, the results showed that there was no statistical difference between the two groups in the average score of inner strength before the intervention (*P* = 0.20), but after the intervention, the average score of inner strength in the intervention group was (97.68 ± 4.53) compared to the control group. (85.46 ± 8.96) had a significant difference (*P* = 0.001) (Table 3).

**Table 3:** Intergroup comparison of Resilience and inner strength in control and intervention groups before and after the intervention

Variable	Groups	Control Mean ± SD	Intervention Mean ± SD	Independent T-Test	*P*-value
Resilience	Before Intervention	49/86 ± 20/70	51.71 ± 4.10	0.46	0.64
	After Intervention	50.07 ± 20.23	67.71 ± 11.86	3.97	< 0.001
Inner strength	Before Intervention	85.50 ± 9.15	88.00 ± 4.69	1.28	0.20
	After Intervention	85.46 ± 8.96	97.68 ± 4.53	6.43	< 0.001

## Discussion

This study aimed to assess the effect of mHealth-CR on the inner Strength and resilience of elderly patients with MI after discharge from the hospital. However, there are a few interventional studies related to this study which have been given in the discussion of the main findings of this study. In the present study, there are two patient outcomes including resilience and inner strength, which have been discussed as follows. The results of the intra-group comparison of the resilience score of the elderly at the present study showed that the mean resilience score before and after the intervention in the control group had no statistically significant difference (*P* = 0.18). Moreover, the present study showed a significant increase in the mean resilience score in the intervention group after the intervention. Vakili et al.‘s study, in which mHealth was not used and face-to-face education was applied, showed that stress management training was effective in the resilience of men with coronary artery disease [92]. There is no study related to mHealth in cardiac rehabilitation patients’ resilience. Education can increase the quality of self-care of the patient and help improve the resilience of the elders. The findings of the present study confirm the effectiveness of the mHealth learning program on elderly patients’ resilience.

In the present study, the comparison between resilience scores of two groups of the elderly showed that before the intervention, the mean resilience score in the control and intervention groups was not statistically different(*P* = 0/64). The study of Naderi et al., Seyed al-Shohdai et al., and Mousavi et al. showed that there was no statistically significant difference between the mean resilience scores of the two groups before the intervention [93–95]. It is obvious that before the start of the intervention, there was no significant difference between the two groups in terms of the investigated variables and background factors. However, after the intervention, the comparison of the mean resilience score between the control and intervention groups showed that this difference is statistically significant (*P* ≤ 0.001). The study of Naderi et al., and Seyed al-Shohdai et al., showed that after the intervention, a statistically significant difference was observed between the mean resilience scores of the intervention and control groups [93, 94]. This means that the mean resilience score after the intervention in the intervention group was significantly higher than the counterparts in the control group. In other words, mHealth can improve the post-discharge MI elderly patients’ resilience scores. This finding is in line with other studies. The results of Mousavi et al.‘s study, examining the effect of patient education and telephone follow-up by a nurse (telenursing) on the resilience of patients with epilepsy, showed that after the implementation of the intervention, the results of the paired t-test showed a statistically significant difference between the overall resilience score and its dimensions in the control and intervention groups, so that the intervention group had relatively higher resilience [95]. The results of Crane’s study showed that the self-reflection training program provided benefits to middle-aged and older adults. Specifically, significant changes in perceived resilience, perceived stress, and positive affect over time emerged for the intervention group compared to the control group [96]. The Irani et al., s’ study showed the Effectiveness of Cognitive-Behavioral Therapy on Resilience in CVDs Patients After Coronary Artery Bypass Graft Surgery [97]. However, in the Crane, et al., Irani et al., and Mousavi et al.,’s studies, mHealth was not used, these findings indicate the positive effect of education and counseling on improving the resilience of patients.

The present study showed that the results of the intra-group comparison of the inner strength score of the elderly in the control group before and after the intervention, there was no statistically significant difference(*P* = 0.71). But there was a significant difference between the mean score of inner strength in the intervention group, before and after the intervention (*P* ≤ 0.001). Also, the results of the intergroup comparison of the inner strength score of the elderly before the intervention showed that there was no statistical difference between the two groups (*P* = 0.20). However, after the intervention, there was a significant difference in the mean score of inner strength in the intervention group and the control group (*P* ≤ 0.001). This means that the mHealth intervention has been effective in increasing the inner strength of the patients. There is no study related to mHealth in cardiac rehabilitation patients’ inner strength. Contrary to our study, the study of Gholami et al. reported that the effect of the self-management support program on the inner strength of cardiac patients was not significant and not effective [59]. The reason for this difference in the findings can be due to the educational face-to-face method, different used inner strength scale, the type of follow-up, and the target group of adult CVDs, i.e. acute myocardial infarction, arrhythmias, heart failure, congenital heart disease, and coronary artery disease (CAD), patients in Gholami’s study compared to cardiac rehabilitation post-MI elderly patients after discharge, and mHealth in our study.

### Strengths and limitations

Low internet speed, which might be disconnected, caused us to conduct online interaction again. In addition, this study was the first study conducted in this area/subject with a relatively moderate sample size.

## Conclusion

The results of this study showed that mHealth as a kind of telenursing has a significant effect on both variables of inner strength and resilience of post-discharge elderly patients after acute myocardial infarction. This means that using mHealth for these patients could increase the inner strength and resilience of the elderly discharged after myocardial infarction. Therefore, through using this method, elderly patients’ self-care ability and quality of life could be increased. It is recommended that healthcare professionals apply this mHealth to supportive care and monitor in CR for discharged elderly patients after Acute MI.

### Electronic supplementary material

Below is the link to the electronic supplementary material.

Supplementary Material 1

Supplementary Material 2

Supplementary Material 3

## Data Availability

The datasets used and/or analyzed during this study are available from the corresponding author.
