# Supplementary material for: The effect of personalized mobile health (mHealth) in cardiac rehabilitation for discharged elderly patients after acute myocardial infarction on their inner strength and resilience
Source: BMC Cardiovasc Disord. 2024 Feb 19;24:116. doi: 10.1186/s12872-024-03791-5 (PMC10877866; doi:10.1186/s12872-024-03791-5)
Supplement: Supplementary file 1 — Supplementary Material 1 [file 12872_2024_3791_MOESM1_ESM.docx]

**Appendix 1:** Content of Personalized Mobile Health (mHealth) in Cardiac Rehabilitation for Discharged Elderly Patients after Acute Myocardial Infarction

| Session | Session Content | Subsidiary Purposes |
| --- | --- | --- |
| First session | Familiarity with the disease and description of its complications. | *How MI is occurred.  *What predictor factors of MI are.  * having the symptoms force you to see a doctor: Feeling pressure or chest pain for more than 15 minutes, dyspnea, fainting or syncope, fast or slow heartbeat, and swelling in the ankle or above.  * reminding the recovery period after a heart attack takes about 1.5-3 months.  *Daily control of weight and blood pressure, treatment if there is high blood pressure  *Addressing personalized patient concerns. |
| Second session | Physical activity.1 | *Regular exercise  *Evaluating the appropriateness of physical activity intensity with speaking test and maximum normal heart rate.  * Doing moderate to intense aerobic daily activities for 30 to 60 minutes.  * Increasing physical activity gradually/Observance of these items in every period of physical activity (30-60 minutes): Warm-up (15 minutes), Main component (10 – 40 minutes), and Cool-down (12 minutes).  *Addressing personalized patient concerns. |
| Third session | Physical activity.2 | *Explanation of how to perform walking, including frequency, duration, and pulse control method within the normal range.  *Stop the activity if you feel a strong heartbeat or feel weak.  *Avoiding sports activities until 2 hours after eating. /Avoiding physical activity up to 2 hours after eating/ Avoiding heavy activities after eating.  *Refraining from exercising during agitation and anxiety and very hot foggy days, especially in cold weather.  *Avoiding driving depending on the severity of the infarction until 2-3 weeks after discharge from the hospital and its initiation in short periods.  *Addressing personalized patient concerns. |
| Fourth session | Nutritional recommendations | * Following a low-salt, low-fat, low-calorie diet, 30 minutes of rest after eating a meal.  * Reducing the consumption of simple sugars in the diet  *Avoiding table salt and adequate fluid intake (reducing the intake of sodium and saturated fats in the diet).  *Increasing the amount of vegetables, fruits, and fish  *Not consuming bulky foods  *It is recommended to use warm milk or light food to improve sleep before going to sleep  *The use of liquid oil e.g., olive, and sunflower oil  *Avoiding eating ready meals, canned foods, tons, strong tea, coffee, and other heavy and high-fat foods such as heart, liver, giblets, offal, and animal fat such as milk, full-fat yogurt, and cream.  *Using onion, garlic, and lemon to replace the salty taste in food.  *Addressing personalized patient concerns. |
| Fifth session | Educational support | *Putting a sublingual pearl before heavy activities and participation in emotional situations  *Avoiding standing too much in the bathroom and sitting on stools and shortening the time of bathing between 15 and 20 minutes  * Adherence to the drug regimen  * Avoiding walking against the wind.  * Establishing regular bowel habits  * Laxative use if necessary  *Addressing personalized patient concerns. |
| Sixth session | Psychosocial support | *Encouragement to talk and communicate with your partner  *Focusing on flirting and romantic touch  *Addressing personalized patient issues  *Lifestyle modification and psychiatric support if the psychologist deems it appropriate  * Avoiding caffeine and smoking in the evening  *Teaching strategies to control depression, stress, and anxiety  *Limiting or stopping using stimulants, such as caffeine, that can cause and exacerbate anxiety.  *See a doctor if you feel depressed  * The importance of maintaining the appropriate pattern of communication with family caregivers.  *Acceptance of limitations  *Avoiding mental stress and nervous tension  *Addressing personalized patient concerns. |
| Seventh session | Educational support | *Weight control and keeping BMI between 18.5 and 24.9  *Getting up slowly (from lying to sitting to standing)  *Avoiding sexual activity when tired and after eating heavy food  *Using pearl sublingual nitroglycerin to prevent heart pain before and after sexual activity  * The best way to travel is by train.  *Avoiding exposure to very cold weather and excessive heat.  *Starting driving from the 4th to the 8th week, Avoiding traveling by plane for up to 6 months. *Addressing personalized patient concerns. |
| Eighth session | Educational support | *Avoiding smoking  *Deep breathing and coughing regularly  *Avoiding activities that cause chest pain, shortness of breath, and fatigue  *Avoiding smoking and alcoholic beverages.  *Avoiding exposure to very cold or very hot weather, including hot showers and hot tubs  *Avoiding long baths in the tub/ Bath duration time is 15-20 minutes.  *Avoiding Valsalva maneuver (straining during bowel movements)  *Addressing personalized patient concerns. |
