# Supplementary material for: The effect of personalized mobile health (mHealth) in cardiac rehabilitation for discharged elderly patients after acute myocardial infarction on their inner strength and resilience
Source: BMC Cardiovasc Disord. 2024 Feb 19;24:116. doi: 10.1186/s12872-024-03791-5 (PMC10877866; doi:10.1186/s12872-024-03791-5)
Supplement: Supplementary file 2 — Supplementary Material 2 [file 12872_2024_3791_MOESM2_ESM.doc]

بسمه تعالي
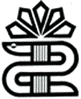


**نام واحد:**

**تاريخ تکميل:**

دانشگاه علوم پزشكي و خدمات بهداشتي و درماني لرستان

**فرم رضايت آگاهانه شرکت درطرح تحقيقاتي مداخله اي**

| **عنوان طرح پژوهشي: تأثیر سلامت شخصی شده موبایل (mHealth) در توانبخشی قلبی بیماران سالمند ترخیص شده پس از انفارکتوس حاد میوکارد بر قدرت درونی و تاب آوری آنها** | **نام مجري طرح:** دکتر شهین سالاروند |
| --- | --- |
| **آقاي/ خانم محترم .....**  **با سلام و آرزوی صحت و سلامتی برای شما**  **شرح پژوهش:**  با توجه به اهمیت ارتقای خودمراقبتی و کیفیت زندگی برای بیماران سالمند ترخیص شده پس از انفارکتوس حاد میوکارد، این مطالعه به منظور تعیین تأثیر توانبخشی مبتنی بر موبایل بر قدرت درونی و تاب‌آوری سالمندان مبتلا به سکته قلبی پس از ترخیص از بیمارستان انجام خواهد شد.  شرکت کنندگان در مطالعه حاضر یک دوره توانبخشی قلبی را گذرانده و سپس قبل و یک ماه بعد از یک دوره یک ماهه توانبخشی قلبی، دو پرسشنامه مذکور را تکمیل خواهند کرد. | |
| **مزاياوفوائد احتمالي** : آموزش های لازم در زمینه توانبخشی قلبی می تواند در ارتقای خودمراقبتی و کیفیت زندگی سالمندان پس از سکته قلبی مفید باشد. | |
| **خسارات وعوارض احتمالي**(اعم ازخسارات روحي،جسمي، اجتماعي،..): خسارتی ندارد. | |
| **جبران خسارات وهزينه ها** (**عدم تحميل هزينه اضافي،نوع بیمه مسئولیت قید شود؛وجود بیمه مسئولیت برای مطالعات دارویی و تجهیزاتی الزامی است.**)  جبران خسارت احتمالی بر عهده مجریان پروژه می باشد. علاوه بر این، همچنین باید توسط شرکت کنندگان اعلام شود. | |
| **نمونه گيري ، دارودرماني يا ساير خدمات(ذکرشود) .** موردی ندارد. | |
| **محرمانه بودن:**  **•** به مشارکت کنندگان اطمینان داده می شود که شرکت در مطالعه کاملا آزاد و داوطلبانه می باشد.  • به مشارکت کنندگان اطمینان داده خواهد شد که برای خروج از پژوهش در هر مرحله آزاد هستند.   - به مشارکت کنندگان اطمینان داده می شود که تجزیه و تحلیل داده ها به صورت کلی انجام می گرددو اصول بی نامی در پیاده کردن، تحلیل و گزارش و نشر اطلاعات انجام خواهد شد. در این رابطه کلیه پرسشنامه ها بدون نام و با کد نگهداری خواهد شد. | |
| **پاسخگويي به پرسشها و تعيين فرد مسئول پاسخگويي**  در صورت هرگونه سوال یا مشکل با دکتر شهین سالاروند مجری اصلی طرح تماس حاصل فرمایید .  شماره تلفن مجری اصلی : 00989161590560  آدرس : دانشیار، مرکز تخقیقات هپاتیت، دانشکده پرستاری مامایی خرم آباد، دانشگاه علوم پزشکی لرستان، ایران. | |
| **حق انصراف درخروج ازمطالعه**  شرکت من درمطالعه کاملاً اختياري است وآزادخواهم بودکه از شرکت درمطالعه امتناع نموده وهرزمان مايل بودم بدون آنكه تغييري درنحوه رفتارپزشک/درمانگريانحوه درمان اينجانب ايجادشودازپژوهش مذکورخارج شوم. | |
| اينجانب ........................... با آگاهي کامل ازموارد فوق رضايت ميدهم که به عنوان يک فرد مورد مطالعه درپژوهش به سرپرستي ( دکتر شهین سالاروند ) شرکت نمايم .  کليه اطلاعاتي که از من گرفته ميشود ونيز نام من محرمانه باقي خواهد ماند ونتايج تحقيقات به صورت کلي ودرقالب اطلاعات گروه مورد مطالعه منتشر ميگردد ونتايج فردي درصورت نياز بدون ذکر نام ومشخصات فردي عرضه خواهد گرديد وهمچنين برائت پزشک يا پزشکان اين طرح را ازکليه اقدامات مذکور دربرگه اطلاعاتي درصورت عدم تقصير درارائه اقدامات اعلام ميدارم.  اين موافقت مانع ازاقدامات قانوني اينجانب درمقابل مجریان طرح درصورتي که عملي خلاف وغير انساني انجام شود نخواهد بود.  امضاءواثرانگشت فردموردپژوهش نام وامضاء مجری اصلی طرح تحقیقاتی  * در صورت بروز هر گونه آسیب و مشکل با شماره تلفن: 06633120172 تماس و یا به آدرس خرم آباد، کمالوند، سایت پردیس دانشگاه علوم پزشکی- معاونت تحقیقات و فناوری، مدیریت تحقیقات دانشگاه مراجعه فرمایید. | |

*اینجانب مجری طرح تحقیقاتی مذکور* ( دکتر شهین سالاروند)  *با آگاهی کامل از کلیه مفاد کدهای حفاظت آزمودنی انسانی در پژوهش های علوم پزشکی که 26 بند می باشد و الزام به رعایت کامل کدهای مذکوراقدام به انجام طرح تحقیقاتی فوق الذکر نموده و تاکید می نمایم که تضمین کننده رعایت این اصول همانا تقوا ،احساس مسئولیت و تعهد اخلاقی اینجانب وهمکاران خواهد بود.*
