# Supplementary material for: The effect of personalized mobile health (mHealth) in cardiac rehabilitation for discharged elderly patients after acute myocardial infarction on their inner strength and resilience
Source: BMC Cardiovasc Disord. 2024 Feb 19;24:116. doi: 10.1186/s12872-024-03791-5 (PMC10877866; doi:10.1186/s12872-024-03791-5)
Supplement: Supplementary file 3 — Supplementary Material 3 [file 12872_2024_3791_MOESM3_ESM.doc]

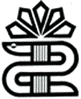


**Code:**

**Date:**

In the name of God

**Informed consent form for participating**

Lorestan University of Medical Sciences

**in an interventional research project**

| **Title:** The Effect of Personalized Mobile Health (mHealth) in Cardiac Rehabilitation for Discharged Elderly Patients after Acute Myocardial Infarction On their Inner Strength and Resilience | **Name of main researcher:**  Dr. Shahin Salarvand |
| --- | --- |
| Dear Sir/Madam………  Greetings and wishing you good health  **Description of the research:**  Given the importance of promoting self-care and quality of life for discharged elderly patients after acute Myocardial Infarction(MI). This study will conduct to determine the effect of mHealth-CR on the inner Strength and resilience of elderly patients with MI after discharge from the hospital.  The participants in the present study will undergo a cardiac rehabilitation course, and then before and one month after a one-month course of cardiac rehabilitation, they will complete two mentioned questionnaires. | |
| **Advantages and potential benefits:**  Necessary training in cardiac rehabilitation can be useful in improving self-care and the quality of life of the elderly after myocardial infarction. | |
| **Potential damages and complications (including mental, physical, social damages,...):**  There are no damages. | |
| **Compensation for damages and expenses (no extra cost should be imposed, the type of liability insurance should be specified; the existence of liability insurance is mandatory for drug and equipment studies.):**  Compensation for any possible damage is the responsibility of the project promoters. moreover, it also must be announced by the participants. | |
| **Laboratory sampling, drug therapy, or other services (to be mentioned):**  None applicable/ There is no case. | |
| **Confidentiality:**  *Participants are assured that participation in the study is completely free and voluntary.  • Participants will be assured that they are free to withdraw from the research at any stage.  • The participants are assured that the data analysis will be done in general and the principles of anonymity will be implemented in the implementation, analysis, reporting, and publication of information. In this regard, all questionnaires will be kept anonymous and coded. | |
| **Answering questions and determining the responsible person for answering:**  In case of any questions or problems, please contact the main project manager with her number: +98 9161590560  Address: Associate Professor, Faculty of Nursing and Midwifery, Lorestan University of Medical Sciences, Khorramabad, Iran. | |
| **The right to withdraw from the study:**  My participation in the study is completely voluntary and I will be free to refuse to participate in the mentioned study and have the right to withdraw from the said study at any time without any change in the behavior of the doctor/therapist/researchers. | |
| I, ......................... fully aware of the above, agree to participate as the study participant in the research under the supervision of Dr. Shahin Salarvand.  All the information that is taken from me, including my name, will remain confidential, and the results of the research will be published in general and in the format of the study group's information, and individual results will be presented if necessary without mentioning the name and individual characteristics, and also the doctor or doctors of this plan will be exempted from all the measures mentioned in the sheet. If there is no fault, I will announce the actions.  This agreement will not prevent me from taking legal action against the plan's executors in the event that an illegal and inhumane act is done.  Signature and fingerprint of the research participant    Name and signature of the principal investigator of the research project  * In case of any damage or problem, call the phone number: 06633120172 or refer to the following address:  Vice President of Research and Technology, University Research Management, Kamalvand campus, Lorestan University of Medical Sciences. | |

I, the executor of the said research project (Dr. Shahin Salarvand), fully aware of all the provisions of the human subject protection code in medical science research, which is 26 clauses, and the obligation to fully comply with the aforementioned codes, have undertaken to carry out the above-mentioned research project, and I emphasize that the guarantee observance of these principles will be the piety, sense of responsibility and moral obligation of me and my colleagues.

*.*
